# Physiological and Metabolic Responses of *Leymus chinensis* Seedlings to Alkali Stress

**DOI:** 10.3390/plants11111494

**Published:** 2022-06-02

**Authors:** Ge Yan, Yujie Shi, Fangfang Chen, Chunsheng Mu, Junfeng Wang

**Affiliations:** Key Laboratory of Vegetation Ecology of the Ministry of Education, Institute of Grassland Science, Jilin Songnen Grassland Ecosystem National Observation and Research Station, Northeast Normal University, Changchun 130024, China; yang674@nenu.edu.cn (G.Y.); shiyj455@nenu.edu.cn (Y.S.); chenff821@nenu.edu.cn (F.C.); wangjf150@nenu.edu.cn (J.W.)

**Keywords:** nucleotides, glutamine synthetase/glutamate synthase cycle, ATP, amino acids, organic acids

## Abstract

To elucidate the physiological and metabolic mechanism of perennial grass responses to alkali stress, we selected *Leymus chinensis* (*L. chinensis*), a salt-tolerant perennial rhizomatous species of the family Poaceae as experimental material. We conducted a pot experiment in a greenhouse and measured the biomass, physiological characteristics, metabonomic, and corresponding metabolites. Our results showed that alkali stress significantly inhibited seedling growth and photosynthesis, which caused ion imbalance and carbon deficiency, but the alkali stress significantly increased the nitrogen and ATP contents. The metabolic analysis indicated that alkali stress markedly enhanced the contents of nucleotides, amino acids, and organic acids, but it decreased soluble sugar contents. Pathway enrichment analysis showed that the glutamine synthetase/glutamate synthase (*GS/GOGAT*) cycle, which was related to nitrogen metabolism, was most significantly affected by alkali stress. The contents of glutamine synthetase (GS) and glutamate synthetase (GOGAT) involved in this pathway were also significantly increased. Our results not only verified the important roles of some amino acids and organic acids in resisting alkali stress, but also further proved that nucleotides and the *GS/GOGAT* cycle related to nitrogen metabolism played critical roles for seedlings in response to alkali stress.

## 1. Introduction

Soil salinization is a severe environmental problem affecting global agriculture. The global area of saline-alkali soil is about 831 million hectares, of which 47% (about 397 million hectares) are saline soil and 53% (about 434 million hectares) are alkaline soil [1]. However, alkali stress with its high pH values is particularly detrimental to plants as compared with salt stress [2]. Previous research has demonstrated that alkali stress can disrupt ion homeostasis, lower water potential, produce excess reactive oxygen species (ROS) and cause high pH stress simultaneously. This induces extensive Na^+^ accumulation in the cytoplasm, inhibiting water absorption by plants, hindering photosynthesis and the synthesis of photosynthetic pigments, destroying the structures of lipids, proteins, DNA and other biological macromolecules in cells, and giving rise to plant growth inhibition or even death [3,4,5,6]. In addition, the high pH around the rhizosphere causes precipitation of mineral ions such as Ca^2+^, Mg^2+^, Cl^−^ and H_2_PO_4_^−^, hence lowering their availability. This high pH around the rhizosphere also destroys membrane structures and disrupts the physiological functions of roots, and ultimately influences plant growth and development [3].

Stress induces plants to produce a large number of biological compounds, including metabolites, which serve an essential part in the metabolic response of plants to stress, and plants can change their physiological functions by regulating the concentrations of metabolites to adapt to stress [7]. Plants have a variety of metabolic adaptation mechanisms to resist adverse environmental conditions, and these mechanisms play a crucial role in plant stress tolerance [8]. Increasing the concentrations of some metabolites, including proline, betaine, γ-aminobutyric acid (GABA), soluble sugars, polyamines, flavonoids, glutathione, and ascorbic acid contributes to increased alkali resistance in plants by eliminating excessive reactive oxygen species (ROS) and regulating osmotic pressure [9,10,11,12]. To mitigate damage from high pH, most plants increase the concentrations of organic acids, however, the nature of this accumulation is species-specific. For instance, under alkali stress, *Puccinellia tenuiflora* (*P. tenuiflora*) mainly accumulates citric acid [13], *Chloris virgata* primarily induces malic acid, and citric acid synthesis [14], while oxalic acid is majorly synthesized in *Kochia scoparia* [15]. Besides metabolites, plants also regulate a great quantity of pathways to minimize the negative effects of alkali stimulation. Studies concerning the metabolic responses of soybean to alkali stress have revealed that energy production from β-oxidation, glycolysis, and the tricarboxylic acid cycle (TCA cycle) plays an important role in this species’ alkali resistance [16]; contrastingly, activating organic acid synthesis to maintain the intracellular ion and pH balance is the main strategy used by wheat, rather than modulating energy metabolism [17]. However, both energy production and organic acid synthesis are the main pathways in the response of corn to alkali stress [3].

*L. chinensis*, is a built-up species distributed in an alkaline meadow in northern China, in the eastern region of the Eurasian steppes. It is often used to improve alkaline soil due to its high feeding value and strong vitality in soils with a pH of over 10 [18]. At present, studies on the response of *L. chinensis* to alkali stress have shown that it can improve alkali tolerance mainly by accumulating small molecular metabolites such as proline, betaine, soluble sugars, organic acids (citric acid, malic acid, succinic acid, acetic acid, oxalic acid) and soluble protein [19,20,21,22,23]. While most studies have only focused on the accumulation of certain metabolites under alkali stress, few systematic studies have been carried out to determine how *L. chinensis* alleviate injury to adapt to stress by regulating the whole metabolic network. We hypothesized that: (1) amino acids, organic acids, and soluble sugars still play important roles during the response of *L. chinensis* seedlings to alkali stress; and (2) the pathways involved in the metabolism of these metabolites will be significantly influenced. Therefore, in the present research, we used ultra-performance liquid chromatography-quadrupole time-of-flight mass spectrometry (UHPLC-triple-TOF-MS) to determine the changes of metabolites in *L. chinensis* seedlings under alkali stress, and then analyzed the related metabolic pathways. The object was to further prove the key metabolites and pathways of *L. chinensis* in response to alkali stress, so as to clarify the internal mechanism of physiological metabolic network regulation.

## 2. Materials and Methods

### 2.1. Plant Materials and Sand Cultures

Seeds of *L. chinensis* were collected from the Grassland Ecosystem Field Station (123°44′ E, 44°40′ N, 167 m asl) of the Northeast Normal University, which is located in the Songnen Grassland of China. The main soil types are saline-alkali soil, aeolian sandy soil, meadow soil, and chernozem [24]. Seeds were harvested in mid-to-late July 2019, which was the mature period of *L. chinensis* seeds. The harvested seeds were stored in a dry and dark environment until the spring of the second year. All seeds were collected with the permission of the head of the Grassland Ecosystem Field Station of the Northeast Normal University, the *L. chinensis* seeds were identified by Prof. Mu. The experiment was conducted in the greenhouse (125°19′ E, 43°51′ N, 236 m asl) of the Northeast Normal University, in Changchun, Jilin, China, in early April 2020. The seedings were sand cultured. River sand with a particle size of about 0.3 mm was sieved and washed, and placed into 12 cm diameter, 10 cm high pots with a base pore (1 cm in diameter). Plump and unified seeds were sterilized on the surface with 2% sodium hypochlorite for 20 min and washed three times with distilled water and then sown in the pots, with 30 seeds per pot. After sowing the seeds, the pots were transferred immediately to a plastic greenhouse. During the experiment, the temperature range was 22–32 °C in the daytime and 14–22 °C at night. The experiment lasted for a total of 27 days, and the seeds started to germinate at around day 7, the emergence rate of each pot was stabilized at around day 12, and we continued to treat the seedings for another 15 days.

### 2.2. Alkali Treatment

Stress treatment was carried out immediately after sowing, with the control group exposed to 0.5× Hoagland nutrient solution and the stress group exposed to alkali-salt (Na_2_CO_3_ and NaHCO_3_, at a 1:1 molar ratio, 45 mmol·L^−1^ Na^+^) mixed with the same volume of solution. Each pot was irrigated at 5:00–6:00 pm every day with 250 mL of the appropriate solution. Individual pots were regarded as single replicates, and four replications were included in each group for a sum of 8 pots. The experiment lasted for 21 days, by which time the third leaf of the seedlings in the control group had fully expanded.

### 2.3. The Measurement of Photosynthetic Parameters

One seedling was selected randomly in each pot to measure the photosynthetic parameters and SPAD values one day before sampling. The second fully expanded leaf was selected. The SPAD values were determined by SPAD chlorophyll meter (SPAD-502, JAPAN), and the net photosynthetic rate (Pn), stomatal conductance (Gs), intercellular CO_2_ concentration (Ci), and transpiration rate (E) were measured with LI-6400XT (Li-Co, Lincoln, NE, USA) portable photosynthesis system at 9:00–11:00 a.m., the photon flux was set at 1200 μmol·m^−2^·s^−1^.

### 2.4. Sample Selection for Physiological Characteristics Determination and Growth Conditions Investigation

At the end of the experiment, we investigated the number of seedlings in each pot. The seedlings which were used to measure photosynthetic parameters were picked as the material to extract metabolites, and they were instantly chilled in liquid nitrogen and saved at −80 °C. Two other seedlings, which had a consistent growth state with the seedlings used to extract metabolites, were selected from each pot. The second fully expanded leaves of these two seedlings were picked to measure the contents of carbon, nitrogen, phosphorus elements, ions, ATP, GS, GOGAT, and metabolites. Half of them were immediately stored in a −80 °C refrigerator to test the results of metabolome and determine the contents of ATP, GS, GOGAT, and metabolites; the other half was dried to determine the contents of ions and elements. All the remaining plants were sampled and washed with distilled water, the surface moisture was wiped off and divided into shoots and roots. The plant height and root length were measured, and then desiccated in an oven at 75 °C to permanent weight to measure the dry weight of shoots and roots.

### 2.5. Measurement of Contents of Ions, Total C, Total N, and Total P

The contents of free cations (K^+^, Na^+^, Ca^2+^, and Mg^2+^) and anions (NO_3_^−^, Cl^−^, SO_4_^2−^ and H_2_PO_4_^−^) in leaves were determined with ion chromatography (DIONEX, Sunnyvale, CA, USA) [25].

The contents of total C and total N in *L. chinensis* leaves were measured with isotope mass spectrometer (Elementary various EL, Langenselbold, UK), and the content of total P was measured with molybdenum antimony resistance detecting method [26].

### 2.6. Determination of the Contents of Osmotic Metabolites, ATP, GS and GOGAT

The contents of 9 organic acids, including oxalic acid, formic acid, tartaric acid, malic acid, lactic acid, acetic acid, maleic acid, citric acid, and succinic acid, and 5 nucleotides, including adenosine 5′-monophosphate (AMP), guanosine 5′- monophosphate (GMP), cytidine 5′-monophosphate (CMP), uridine 5′-monophosphate (UMP) and inosine 5′-monophosphate (IMP) in *L. chinensis* leaves were measured with high-performance liquid chromatography [27,28], the total contents of soluble sugar, amino acids, ATP, GS, and GOGAT were measured with ELISA kits (Shanghai Enzyme-linked Biotechnology Co., Ltd., Shanghai, China), soluble sugar was determined by anthrone colorimetric method, amino acid was determined by ninhydrin colorimetric method, ATP was determined by phosphomolybdic acid colorimetric method, and GS and GOGAT were determined by double antibody sandwich method.

### 2.7. Metabolite Extraction and Detection

Sample extraction and detection were carried out according to the method of Chen et al. with some modifications [29]. At first, approximately 500 mg of each lyophilized sample was transferred to a 2 mL Eppendorf tube, after which 1000 µL of methanol/acetonitrile/water (2:2:1, *v*/*v*/*v*) containing 20 µL of ribitol (1 mg/mL) was added to each tube as an internal standard. Samples were scrolled for 30 s and uniformed in a ground powder system (JXFSTPRP-24, Jinxin Technology, Shanghai, China) at 45 Hz for 4 min, followed by supersonic processing in ice water for 5 min. The above-described procedure was lastly repeated 3 times before culturing the samples at −20 °C for 1 h. The tubes were later subjected to centrifugation (Heraeus Fresco17 centrifuge, Thermo Scientific, Waltham, MA, USA) at 13,000 rpm for 15 min at 4 °C. Approximately 0.4 mL of the supernatant was decanted into a new Eppendorf tube and the supernatant was dried in a vapor concentrator. Afterward, each sample was dissolved in 600 µL of acetonitrile/water (1:1, *v*/*v*), vortexed for 30 s, and then sonicated for 5 min (if not dissolved, extend the time appropriately). The samples were centrifuged again according to the above method. In the end, roughly 60 μL of supernatant was filtered through a 0.22 μm filter and dispensed into a new 2 mL LC/MS glass bottle. Ten μL of each sample was extracted and intermixed as a QC sample for assay. A UHPLC device (Infinity 1290, Agilent Tech., Santa Clara, CA, USA) and a UPLC BEH Amide column (1.7 μm, 2.1 × 100 mm, Waters Corp., Milford, MA, USA) in combination with a triple TOF 6600 system (Q-TOF, AB Sciex, Concord, ON, Canada) for LC-MS/MS analysis. The mobile phase consisted of 25 mM NH4OAc and 25 mM NH4OH in water (pH = 9.75) and pure acetonitrile. The step elution program was as follows: 0 min, 95% B; 0.5 min, 95% B; 7 min, 65% B; 8 min, 40% B; 9 min, 40% B; 9.1 min, 95% B; 12 min, 95% B; the injection volume was 0.5 μL. MS/MS profiles were obtained using triple TOF-MS based on information acquisition technique (IDA). MS databases were continuously collected and evaluated by acquisition software with full scan survey (Analyst TF 1.7, AB Sciex), and MS/MS spectra were acquired depending on preset parameters. Within each cycle, precursor ions with strengths above 100 were picked and then fragmented at 30 V collision energy (CE) (15 MS/MS events every 50 ms). The electrospray ionization (ESI) source was set to 60 PSI nebulizer pressure, 60 PSI auxiliary gas pressure, 30 PSI air curtain pressure, 650°C source temperature, and ion spray voltage float (ISVF) of 5000 or −4000 V in positive and negative patterns, respectively.

### 2.8. Data Processing and Multivariate Data Analysis

For the raw data of the UPLC-Triple TOF-MS, preprocessing consists of the steps: first, removing any features with a minimum of 50% missing values; second, replacing the missing values using a small value of half of the minimum positive value of the raw data; finally, normalization was further performed using the total ion current (TIC) or each sample. In addition, the pre-processed data were used to scan the local database for material details, creating a table including metabolite names and scores, matched patterns, the mass-to-charge ratio (*m*/*z*), retention time, and MS intensity for each sample. During the analysis of the QC samples, ions with a relative standard deviation (RSD) < 30% were selected for further analysis using univariate and multivariate methods.

MetaboAnalyst 4.0 (https://www.metaboanalyst.ca/, accessed on 15 December 2021) was used to conduct principal component analysis (PCA), orthogonal projection to latent structure discriminant analysis (OPLS-DA), and pathway analysis after the primary data processing. In the PCA analysis, we performed log transformation and centrally formalized data, followed by automodeling analysis. To remove orthogonal variables in metabolites that were not related to classification variables and to gain more credible metabolite information on the differences between groups, we applied OPLS-DA to retrieve the largest information on metabolic changes under alkali stress and the importance of metabolites causing the changes (VIP values). The differential metabolites were identified using the Student’s *t*-test and the importance value (VIP > 1). Differential metabolites were identified according to the Oryza sativa L. database in MetaboAnalyst 4.0 and the PubChem database (https://pubchem.ncbi.nlm.nih.gov/, accessed on 15 December 2021). The KEGG database was then used to annotate the pathways of different metabolites so as to find key pathways associated with the differential metabolites.

### 2.9. Statistical Analysis

Photosynthetic pigments, metal elements, and inorganic anions were statistically analyzed using SPSS 20 (SPSS Institute, Cary, NC, USA). Significant differences between the control and alkali treatments were tested by *t*-test.

## 3. Results

### 3.1. Changes in Growth Parameters of L. chinensis under Alkali Stress

Alkali stress-induced growth inhibition was significant in *L. chinensis* seedlings, including the significant decreases in the germination rate, shoot height, root length, shoot and root dry weights and leave number (*p* < 0.05). Meanwhile, the inhibition effects were more evident in shoots than in roots. This was supported by the greater decline in height (0.13-fold) and dry weight (0.17-fold) in shoots than in roots (length 0.09-fold and dry weight 0.12-fold) (Table 1).

### 3.2. Changes in Photosynthetic Parameters of L. chinensis under Alkali Stress

Alkali stress-induced photosynthesis inhibition was significant in *L. chinensis* seedlings, including the significant decreases in the Pn, Ci, Gs, E, and SPAD values (*p* < 0.05) (Table 2).

### 3.3. Changes in Ions and Elements of L. chinensis under Alkali Stress

The Na^+^ content was significantly increased (*p* < 0.01), while the contents of K^+^ and Ca^2+^ were significantly decreased under alkali stress (*p* < 0.05); In contrast, the contents of NO_3_^−^ and H_2_PO_4_^−^ were significantly decreased (*p* < 0.05), while the effects of alkali stress on Mg^2+^, Cl^−^ and SO_4_^2−^ were not significant. Alkali stress had inconsistent effects on the elemental contents of seedlings. The carbon content decreased significantly (*p* < 0.05), the nitrogen content increased significantly (*p* < 0.05), while the phosphorus content did not change (Table 3).

### 3.4. Changes in Metabolites in L. chinensis under Alkali Stress

**Figure 1:**
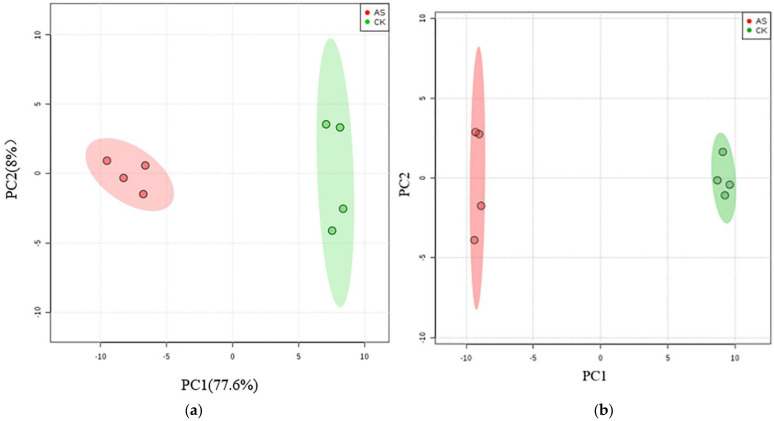
PCA (**a**) and OPLS−DA (**b**) analyses of metabolic profiles of *L. chinensis* treated with and without alkali stress.

The results of the PCA score plot showed that the samples were clearly separated by two principal components, which explained about 86% of the variability (Figure 1a). The major contributors to PC1 were N-acetyl-l-aspartic acid, 2′-deoxycytidine 5′-monophosphate (dCMP), glutamine, which were all down-regulated under alkali stress. In contrast, the contribution of metabolites to PC2 included thymidine, deoxyguanosine, deoxycytidine, cytidine 5′-monophosphate (CMP), and cytosine, which are all nucleotides and up-regulated under alkali stress (Supplementary Table S1). OPLS-DA was used to further analyze the data from the two groups, with R^2^ and Q^2^ representing the fit and the predictive capability of the model, respectively, and both of these values indicated the reliability of the model. The results further showed the obvious separation between the two groups with good model quality (R^2^ = 0.79, Q^2^ = 0.98) (Figure 1b).

**Figure 2:**
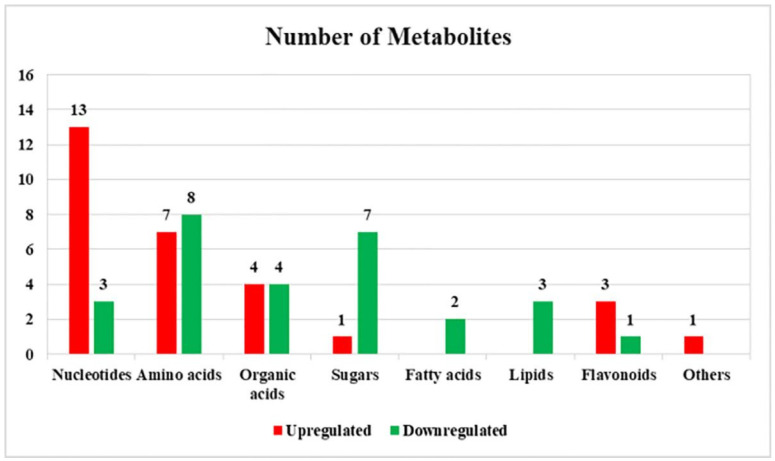
The numbers of upregulated and downregulated differential metabolites in *L. chinensis* under alkali stress.

According to the standard of VIP > 1 and *p* < 0.05, 57 differential metabolites were screened including 16 nucleotides, 15 amino acids, 8 organic acids, 8 sugars, 2 fatty acids, 3 cholines, 4 flavonoids, and 1 other compound. Among them, 29 metabolites had greater content levels under alkali stress and 28 metabolites had greater accumulation levels under control (Figure 2).

Among various differential metabolites, the contents of 13 nucleotides including deoxycytidine, 5-methylcytosine, and cytidine were significantly increased; only three nucleotides including 2′-deoxycytidine diphosphate (dCDP), 2′-deoxycytidine 5′-monophosphate (dCMP) and adenosine monophosphate (AMP) were significantly decreased; the contents of amino acids including ACC, cystine, asparagine, tryptophan, o-acetyl--l-serine, arginine, and GABA were increased (listed from largest to smallest increase), while the accumulation of other amino acids including N-acetyl-aspartic acid, glutamine, glutamate, serine, phenylalanine, homoserine, threonine, and tyrosine was reduced (listed from largest to smallest decrease); and the contents of ascorbic acid, galactarate, tartaric acid, and glucarate were increased (listed from largest to smallest increase), while alpha-ketoglutaric acid, succinic acid, glyceric acid and malic acid were decreased (listed from largest to smallest decrease). The contents of seven soluble sugars, including xylose, sucrose, and N-acetyl-glucosamine-1-phosphate, etc., decreased significantly, only arabinose increased significantly; the contents of two fatty acids, including stearic acid and oleic acid, and three cholines, including choline, phosphocholine, and phosphatidylcholine, were all decreased; among the flavonoids, the vitexin content decreased, whereas the contents of kaempferol 3-o-rutinoside, apioside, and apigenin all increased under alkali stress (Supplementary Table S2).

Among the up-regulated metabolites, the order of those with a fold change greater than two from high to low were deoxycytidine, ACC, 5-methylcytosine, cytidine, deoxyadenosine, cytosine, cystine, and thymidine, among six of which were nucleotides and two were amino acids; in the down-regulated metabolites, the order of those with fold change less than −2 from low to high were N-acetyl-aspartic acid and dCMP, with one nucleotide and one amino acid (Supplementary Table S2).

### 3.5. Changes to Metabolic Pathways in L. chinensis under Alkali Stress

**Figure 3:**
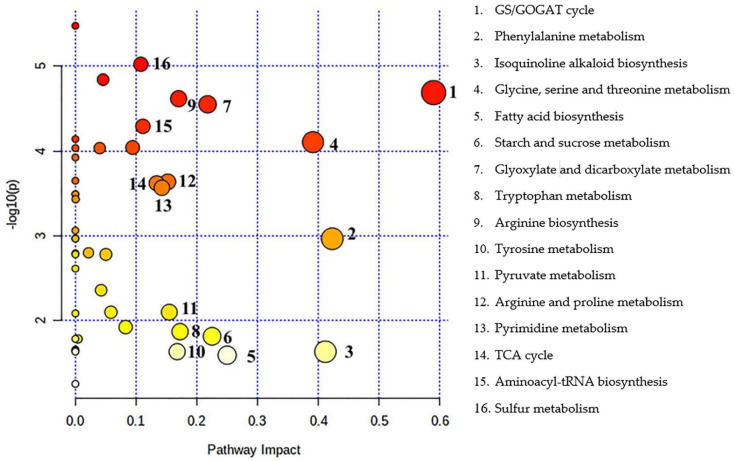
Enrichment analysis of metabolic pathways in *L. chinensis* seedlings. Circle color from shallow to deep represents *p*−value from large to small, circle. radius from small to large represents impact value from small to large. The deeper the circle color, the greater the radius represents the greater the impact of alkali stress on the pathway. The circle closer to the upper right corner of the pathway diagram represents the more significant effect of alkali stress on this metabolic pathway.

Pathway enrichment analysis showed 16 metabolic pathways that were significantly affected by alkali stress, including GS/GOGAT cycle, phenylalanine metabolism, isoquinoline alkaloid biosynthesis, glycine, serine, and threonine metabolism, cutin, suberine, and wax biosynthesis, starch and sucrose metabolism, glyoxylate and dicarboxylate metabolism, tryptophan metabolism, arginine biosynthesis, tyrosine metabolism, pyruvate metabolism, arginine, and proline metabolism, pyrimidine metabolism, TCA cycle, Aminoacyl-tRNA biosynthesis, and sulfur metabolism, among them, the *p*-value of *GS/GOGAT* cycle was the smallest (*p* < 0.01), and the impact value was the largest (about 0.59), which was the most significant pathway affected by alkali stress (Figure 3; Table 4).

### 3.6. Changes of Four Metabolites, Two Enzymes, and ATP in L. chinensis under Alkali Stress

Separate measurements of the total contents of nucleotides, amino acids, organic acids, and soluble sugars identified by metabolomic analysis showed that organic acids, amino acids, and nucleotides were increased significantly (*p* < 0.05), while soluble sugars were decreased significantly under alkali stress (*p* < 0.01); measurements of the two most important enzymes involved in the *GS/GOGAT* cycle, which were the most significantly affected pathway, showed that both GS and GOGAT increased significantly under alkali stress (*p* < 0.01), the results of the ATP assay showed that it increased significantly under alkali stress (*p* < 0.01) (Table 5).

## 4. Discussion

### 4.1. Physiological Response of L. chinensis Seedlings under Alkali Stress

Previous studies have shown that alkali stress induces a large accumulation of Na^+^ and causes ion toxicity. Alkali stress can reduce the accumulation of photosynthetic pigments and causes the synthesis of photosynthetic products such as soluble sugars to be blocked by hindering the photosynthetic carbon fixation capacity of plants, resulting in energy deficiency and decreased ATP content in plants. The high pH can cause the large precipitation of metal ions such as Ca^2+^ and Mg^2+^, which inhibits the uptake of inorganic anions such as NO_3_^−^ and H_2_PO_4_^−^ and reduces the utilization of nutrients such as N and P, resulting in nutrient deficiency. These can cause plant growth inhibition and even death [3,4,5,6,17]. However, our results concluded that the contents of nitrogen and ATP in *L. chinensis* seedlings increased significantly under alkali stress, Guo et al. [30] also found that the total nitrogen content of *P. tenuiflora* seedlings increased under low alkali stress (<60 mmol/L). This may be a specific way for this species to respond to stress by enhancing nitrogen fixation to promote the accumulation of small molecule nitrogenous metabolites for improving osmoregulation, and it can simultaneously ensure sufficient energy supply to mitigate stress injury.

### 4.2. Metabolites Responding to Alkali Stress

Previous studies have suggested that when plants are subjected to salt and alkali stress, their contents of soluble sugars [19], organic acid like malic acid [19,20] and amino acids such as proline [19,20,21,22,23] increased, which were the main organic solutes against stress. However, our results showed that among the eight significantly different soluble sugars, the contents of seven sugars, such as sucrose and glucose-6p, were significantly reduced. The proportions of eight organic acids and fifteen amino acids significantly increased or decreased were basically the same. Ascorbic acid was the organic acid whose content was significantly increased, while the contents of four organic acids like malic acid, were significantly reduced. The seven amino acids whose contents were significantly increased were ACC, cystine and asparagine, etc., but no significant change in proline concentration was found. The results of the metabolites also showed that the content of soluble sugar decreased significantly, while the contents of organic acids and amino acids increased significantly. These results indicated that soluble sugars were not the responsive metabolites of *L. chinensis* seedlings to resist alkali stress, which was consistent with previous studies on metabolic responses of wheat seedlings to alkali stress [17].

Although organic acids and amino acids were also the main metabolites of *L. chinensis* seedlings in response to alkali stress, the specific types of responses were different from those of previous studies, which might be related to the fact that *L. chinensis* seedlings in this study were at the seedling stage, with only three young leaves, small leaf area, and weak photosynthetic and alkali tolerance, Guo et al. [17] found that most organic acid contents decreased under alkali stress in wheat seedlings, and may be related to carbon source deficiency caused by alkali stress inhibiting photosynthesis of seedlings. Yamani et al. [31] also found that proline content increased with the extension of the olive growth period under drought stress, and early accumulation was not significant. Our results also showed that two fatty acids concentrations were significantly decreased, which was consistent with previous studies on metabolic changes of wheat seedlings under salt stress [32], suggesting that plant cell membrane was damaged under stress [33].

Nucleotides, as small molecular metabolites of important life activities such as plant genetics and energy metabolism, have been reported to accumulate in previous studies on the response of *P. tenuiflora* seedings to alkali stress [34]. Our result showed that most nucleotides’ contents increased, and nucleotides accounted for the largest proportion of metabolites in the accumulation of *L. chinensis* seedlings induced by alkali stress. This combined with the result that nucleotide total content also increased significantly under alkali stress, therefore, nucleotide was another kind of the most important metabolite of *L. chinensis* seedlings in response to alkali stress. The accumulation of deoxynucleosides like deoxycytidine, deoxyadenosine, and deoxyguanosine may be related to the repair of damaged DNA under stress [35]. In addition, studies have shown that salt stress promotes the biosynthesis of purine and pyrimidine nucleotides and provides ATP energy for plants [36], In *L. chinensis* seedlings, the contents of metabolites involved in the TCA cycle and glycolysis significantly decreased under alkali stress, indicating that alkali stress inhibited the energy production pathway, resulting in the decrease of ATP and other energy substances, Zang et al. [37] also found that the energy supply correlated pathways, including glycolysis and TCA cycle, were impacted by salt and alkali stress in *Suaeda corniculata* seedings. However, the results of ATP content showed opposite results, with a significant increase in the ATP content of seedlings under alkali stress. Therefore, it may be necessary to accumulate a large quantity of nucleotides to participate in the synthesis of ATP for seedling growth and energy supply. The versatility of nucleotides makes them occupy a major position in the alkali resistance of *L. chinensis* seedlings.

### 4.3. Metabolic Pathways Responding to Alkali Stress

Among 16 pathways significantly affected by alkali stress, *GS/GOGAT* cycle was most significantly affected, and the contents of related intermediates, including N-acetyl-aspartic acid, glutamate, and glutamine, were significantly reduced under alkali stress. In contrast, the contents of two key enzymes (GS and GOGAT) involved in this pathway were significantly increased under alkali stress, suggesting that alkali stress activates this pathway in seedlings, our results were consistent with those of Hussain et al. [38] on the activities of enzymes related to nitrogen metabolism in tomato seedlings under salt stress. Because *GS/GOGAT* cycle is the main pathway of nitrogen assimilation in higher plants, the pathway may be induced by alkali stress to supply nitrogen sources for seedlings to synthesize small nitrogen-containing osmotic solutes such as nucleotides and amino acids, the enhancement of amino acid metabolism with special functions in seedlings may help to improve their alkali tolerance [39]. These amino acid metabolic pathways include arginine and proline metabolism and arginine biosynthesis, etc., as the principal pathways for the synthesis of main osmotic adjustment substances, such as polyamines and GABA, they play a major role in improving plant stress tolerance [8]. Yang et al. [40] found that the salt tolerance of *Crossostephium chinensis* seedlings can be improved by inducing the accumulation of metabolites related to these pathways and enhancing the activity of metabolic enzymes.

As mentioned above, the study on differential metabolites found that nucleotide was another kind of critical metabolite for *L. chinensis* seedlings to respond to alkali stress. Pathway analysis also found that the pyrimidine metabolism was significantly affected by alkali stress, and the nucleotide concentrations significantly increased, indicating that alkali stress promoted pyrimidine metabolism. This may be a special way for *L. chinensis* seedlings to adapt to alkali stress, by inducing pyrimidine metabolism to promote ATP synthesis and improve alkali resistance under the condition of insufficient energy.

**Figure 4:**
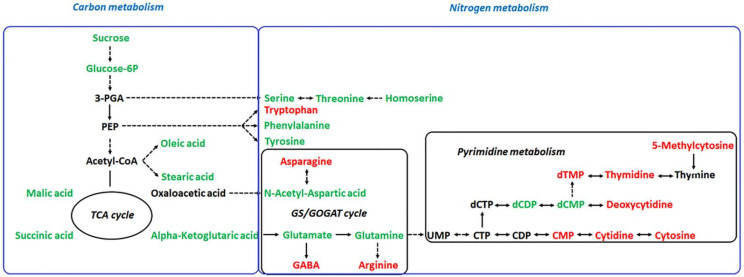
Changes in metabolic pathways of *L. chinensis* seedlings responding to alkali stress. Red-marked metabolites indicated that the metabolite concentration increased significantly under alkali stress (*p* < 0.05). Green-marked metabolites indicated that the metabolite concentration decreased significantly under alkali stress (*p* < 0.05). Black-marked metabolites showed no significant change.

Sugar metabolism, TCA cycle, and fatty acid metabolism provide a large amount of energy for plant growth, which is closely associated with the salt and alkali resistance of plants [3,16,32], but this study found that the contents of most related metabolites in these pathways were significantly decreased under alkali stress, indicating that alkali stress inhibits the energy metabolism of *L. chinensis* seedlings, which also suggested that energy supply is not the main way for *L. chinensis* seedlings to resist alkali stress. The same results were found in the study of metabolic changes of wheat and *Haloxylon salicornicum* seedlings under salt and alkali stress [17,33]. Since these three pathways are major components of carbon metabolism, metabolic activity is closely related to carbon fixation efficiency [17,39], so these pathways may be related to the low photosynthetic carbon sequestration capacity of seedlings, resulting in insufficient carbon sources. Pathways diagrams are portrayed in Figure 4.

## 5. Conclusions

Alkali stress hindered the photosynthetic carbon fixation capacity of *L. chinensis* seedlings, resulting in the shortage of carbon sources, decreased the contents of soluble sugars and organic acids, but induced *GS/GOGAT* cycle to accelerate nitrogen assimilation and supply nitrogen sources for seedlings to synthesis small nitrogen-containing osmotic solutes, such as nucleotides and amino acids, to improve osmoregulation and provide ATP. Besides amino acids and organic acids, nucleotides and the *GS/GOGAT* cycle related to nitrogen metabolism also played critical roles for seedlings in response to alkali stress. In order to fully reveal the physiological and metabolic responses of *L. chinensis* to alkali stress, we suggest further studies on the physiological and metabolic responses of *L. chinensis* to alkali stress for other important phenological periods in the future.

## Figures and Tables

**Table 1:** The growth parameters of *L. chinensis* under normal and alkali conditions. The germination rate was calculated using the formula (the number of seedlings per pot / 30) × 100%. The fold changes were calculated using the formula log_2_^(alkali/control)^. Significant differences between control and alkali stress were defined with the *t*-test and labeled as *p* < 0.05 and *p* < 0.01.

Growth Parameters	log_2_^(AS/CK)^	*p*-Value
Germination rate (%)	−0.22	<0.05
Shoot height (cm)	−0.13	<0.01
Root length (cm)	−0.09	<0.05
Dry weight of shoots (g)	−0.17	<0.01
Dry weight of roots (g)	−0.12	<0.01
Leaf number	0.25	<0.05

**Table 2:** The photosynthetic parameters of *L. chinensis* under normal and alkali conditions. The fold changes were calculated using the formula log_2_^(alkali/control)^. Significant differences between control and alkali stress were defined with the *t*-test and labeled as *p* < 0.05 and *p* < 0.01.

Photosynthesis Parameters (μmol CO_2_ ·m^−2^·s^−1^)	log_2_^(AS/CK)^	*p*-Value
Pn	−0.29	<0.05
Gs	−0.18	<0.01
Ci	−0.11	<0.05
E	−0.19	<0.01
SPAD value	−1.18	<0.05

**Table 3:** The contents of ions and elements of *L. chinensis* under normal and alkali conditions. The fold changes were calculated using the formula log_2_^(alkali/control)^. Significant differences between control and alkali stress were defined with the *t-test* and labeled as *p* < 0.05 and *p* < 0.01.

Ion and Element Contents		log_2_^(AS/CK)^	*p*-Value
Ion content (μmol·g^−1^ DW)	Na^+^	0.35	<0.01
	K^+^	−0.27	<0.05
	Ca^2+^	−0.15	<0.05
	Mg^2+^	−0.10	NS
	Cl^−^	0.04	NS
	NO_3_^−^	−0.05	<0.05
	H_2_PO_4_^−^	−0.07	<0.01
	SO_4_^2−^	0.12	NS
Element content (mg·g^−1^)	C	−0.24	<0.05
	N	0.15	<0.05
	P	−0.02	NS

**Table 4:** Pathway enrichment analysis depicting significantly altered metabolic pathways in *L. chinensis* in response to alkali stress.

	Total Compound	Hits	Raw *p*	Log (*p*)	Holm Adjust	FDR	Impact
GS/GOGAT cycle	22	5	0.000020	4.6893	0.000859	0.000211	0.58992
Phenylalanine metabolism	12	1	0.001087	2.9638	0.025850	0.002038	0.42308
Isoquinoline alkaloid biosynthesis	6	1	0.023442	1.6300	0.155050	0.024532	0.41176
Glycine, serine, and threonine metabolism	33	7	0.000079	4.1039	0.002913	0.000347	0.39130
Fatty acid biosynthesis	14	1	0.025780	1.5887	0.155050	0.026366	0.25000
Starch and sucrose metabolism	22	2	0.015432	1.8116	0.154320	0.019289	0.22509
Glyoxylate and dicarboxylate metabolism	29	7	0.000028	4.5510	0.001125	0.000211	0.21779
Tryptophan metabolism	23	1	0.013612	1.8661	0.149740	0.017502	0.17241
Arginine biosynthesis	18	4	0.000024	4.6172	0.000990	0.000211	0.16991
Tyrosine metabolism	18	1	0.023442	1.6300	0.155050	0.024532	0.16757
Pyruvate metabolism	22	1	0.008012	2.0963	0.120170	0.011266	0.15462
Arginine and proline metabolism	28	2	0.000231	3.6360	0.007190	0.000677	0.15223
Pyrimidine metabolism	38	8	0.000272	3.5653	0.007891	0.000720	0.14246
Citrate cycle (TCA cycle)	20	3	0.000241	3.6186	0.007219	0.000677	0.13384
Aminoacyl-tRNA biosynthesis	46	9	0.000051	4.2909	0.001996	0.000329	0.11111
Sulfur metabolism	15	3	0.000009	5.0239	0.000416	0.000211	0.10774

**Table 5:** The contents of metabolites, ATP, GS, and GOGAT of *L. chinensis* under normal and alkali conditions. The fold changes were calculated using the formula log_2_^(alkali/control)^. Significant differences between control and alkali stress were defined with the *t*-test and labeled as *p* < 0.05 and *p* < 0.01.

Metabolites	log_2_^(AS/CK)^	*p*-Value
Amino acid	0.13	<0.01
Organic acid	0.04	<0.01
Sugar	−0.13	<0.01
Nucleotide	0.19	<0.05
ATP	0.06	<0.01
GS	0.10	<0.01
GOGAT	0.11	<0.01

## Data Availability

Research data are not shared.

## Supplementary Materials

The following supporting information can be downloaded at: https://www.mdpi.com/article/10.3390/plants11111494/s1, Table S1: The contribution of metabolites in seedling leaves to the first principal component (PC1) and the second principal component (PC2); Table S2: Relative contents and fold changes of metabolites in seedling leaves under alkali treatment.
