# Supplementary material for: Physiological and Metabolic Responses of Leymus chinensis Seedlings to Alkali Stress
Source: Plants (Basel). 2022 Jun 2;11(11):1494. doi: 10.3390/plants11111494 (PMC9182738; doi:10.3390/plants11111494)
Supplement: Supplementary file 1 [file plants-11-01494-s001.zip › plants-1749576-supplementary.pdf]

Table S1. The contribution of metabolites in seedling leaves to the first principal component (PC1) and the second principal component (PC2).

Metabolite Name	PC1	PC2
thymidine	-0.12	0.28
deoxyguanosine	-0.09	0.23
deoxycytidine	-0.17	0.21
CMP	-0.09	0.16
cytosine	-0.12	0.15
5-methylcytosine	-0.13	0.13
tartaric acid	-0.09	0.12
deoxyadenosine	-0.12	0.09
betaine aldehyde	-0.09	0.08
glucose-6p	0.09	0.07
arginine	-0.06	0.07
fucose	0.09	0.06
xylose	0.10	0.04
stearic acid	0.10	0.04
oleic acid	0.06	0.04
guanosine	-0.05	0.04
glutamate	0.10	0.03
glyceric acid	0.09	0.03
serine	0.10	0.02
GlcNAc-1p	0.10	0.02
phosphatidylcholine	0.10	0.02
sucrose	0.08	0.02
dCDP	0.07	0.02
homoserine	0.07	0.02
malic acid	0.07	0.02
phosphorylcholine	0.07	0.02
phosphatidylcholine	0.07	0.02
ascorbic acid	-0.11	0.02
glutamine	0.11	0.01
threonine	0.06	0.01
cytidine	-0.12	0.01
N-acetyl-l-aspartic acid	0.18	0.00
choline	0.06	0.00
dTMP	-0.09	0.00
galactarate	-0.1	0.00
dCMP	0.14	-0.01
phenylalanine	0.08	-0.01
kaempferol 3-o-rutinoside	-0.07	-0.01
succinate	0.10	-0.02
tyrosine	0.06	-0.02
apigenin	-0.05	-0.02
glucosamine	0.08	-0.03
3'-AMP	-0.06	-0.03
arabinose	-0.07	-0.03
dAMP	-0.09	-0.03
alpha-ketoglutaric acid	0.10	-0.04

o-Acetyl-l-serine	-0.07	-0.04
ACC	-0.12	-0.04
glucarate	-0.05	-0.06
vitexin	0.11	-0.08
GlcNAc	0.09	-0.09
AMP	0.11	-0.11
GABA	-0.04	-0.11
allantoin	-0.07	-0.12
cystine	-0.12	-0.15
tryptophan	-0.09	-0.18
asparagine	-0.09	-0.21

CMP, cytidine 5'-monophosphate; dTMP, deoxythymidine 5'-phosphate; dAMP, 2'-deoxyadenosine 5'-monophosphate; 3'-AMP, adenosine 3'-monophosphate; dCDP, 2'-deoxycytidine diphosphate; dCMP, 2'-deoxycytidine 5'-monophosphate; AMP, adenosine monophosphate; ACC, 1-aminocyclopropane-1-carboxylic acid; GABA, 4-acetamidobutyric acid; GlcNAc, N-acetyl-D-glucosamine; GlcNAc-1p, N-acetyl-glucosamine-1-phosphate.

Table S2. Relative contents and fold changes of metabolites in seeding leaves under alkali treatment.

**Compound Name**	**Platform**	**Relative Concentration**		**Fold Change**	**P Value**
		**CK**	**AS**	**Log $_{2}$ $^{(AS/CK)}$**	
Nucleotide					
deoxycytidine	pos	0.12	1.94	3.97	<0.01
5-methylcytosine	pos	0.2	1.06	2.43	<0.01
cytidine	pos	0.14	0.7	2.38	<0.05
cytosine	pos	0.24	1.16	2.30	<0.01
thymidine	neg	0.46	2.14	2.23	<0.05
dTMP	neg	0.04	0.1	1.55	<0.05
CMP	pos	0.19	0.55	1.49	<0.01
deoxyadenosine	pos	2.14	10.87	2.34	<0.05
dAMP	neg	0.01	0.03	1.52	<0.05
deoxyguanosine	neg	0.41	1.13	1.45	<0.01
allantoin	neg	1.22	2.59	1.08	<0.05
3'-AMP	neg	0.04	0.06	0.84	<0.05
guanosine	pos	0.03	0.05	0.73	<0.05
dCDP	neg	0.03	0.02	-0.45	<0.05
dCMP	pos	0.21	0.05	-2.05	<0.01
AMP	neg	0.19	0.07	-1.37	<0.05
Flavonoid					
kaempferol 3-o-rutinoside	pos	0.13	0.27	1.03	<0.01
apioside	neg	0.18	0.36	0.98	<0.01
apigenin	pos	0.49	0.82	0.76	<0.05
vitexin	pos	0.15	0.06	-1.34	<0.05
Others					
betaine aldehyde	pos	0.28	0.80	1.51	<0.05
Amino acid					
ACC	pos	0.02	0.11	2.60	<0.05
cystine	pos	0.01	0.06	2.26	<0.01

asparagine	pos	0.20	0.60	1.62	<0.01
tryptophan	pos	0.02	0.05	1.37	<0.01
o-acetyl--l-serine	neg	0.02	0.05	1.03	<0.01
arginine	pos	0.05	0.09	0.84	<0.05
GABA	pos	2.42	3.83	0.66	<0.01
tyrosine	pos	0.21	0.18	-0.26	<0.05
threonine	neg	0.53	0.44	-0.27	<0.05
homoserine	pos	3.00	2.28	-0.39	<0.01
phenylalanine	pos	0.59	0.43	-0.45	<0.01
serine	pos	0.20	0.10	-1.02	<0.01
glutamate	neg	0.28	0.13	-1.16	<0.05
glutamine	neg	2.78	1.15	-1.28	<0.01
N-acetyl-l-aspartic acid	neg	0.27	0.02	-3.69	<0.01
Carbohydrate					
arabinose	pos	0.24	0.49	1.00	<0.05
glucosamine	neg	0.25	0.16	-0.67	<0.05
fucose	pos	0.05	0.03	-0.77	<0.05
GlcNAc	neg	0.05	0.03	-0.84	<0.05
glucose-6p	neg	0.08	0.04	-0.93	<0.05
GlcNAc-1p	neg	0.06	0.03	-1.01	<0.01
sucrose	neg	7.00	3.19	-1.14	<0.01
xylose	pos	0.41	0.17	-1.25	<0.01
Organic acid					
ascorbic acid	neg	0.10	0.36	1.90	<0.01
galactarate	neg	0.51	1.65	1.70	<0.05
tartaric acid	neg	0.07	0.19	1.35	<0.01
glucarate	neg	0.06	0.11	0.78	<0.05
malic acid	neg	7.22	5.64	-0.36	<0.01
glyceric acid	neg	0.08	0.05	-0.81	<0.01
succinate	neg	1.17	0.64	-0.88	<0.01
alpha-ketoglutaric acid	neg	0.03	0.01	-1.20	<0.05
Fatty acids					
oleic acid	pos	1.20	1.06	-0.17	<0.05
stearic acid	pos	0.14	0.06	-1.16	<0.01
Lipid					
choline	pos	19.96	16.20	-0.30	<0.05
phosphorylcholine	pos	1.72	1.27	-0.44	<0.05
phosphatidylcholine	pos	2.13	0.98	-1.12	<0.05

The relative concentration of each metabolite is the average of UHPLC-Triple-TOF-MS data from four biological replicates. The fold changes were calculated using the formula $log_2^{(AS/CK)}$. CK: Control, AS: Alkali stress. Relative contents values were increased 10 times in each treatment. Significant differences between control and alkali stress were determined with the *T-test* and marked as $P < 0.05$ and $P < 0.01$. dTMP, deoxythymidine 5'-phosphate; CMP, cytidine 5'-monophosphate; dAMP, 2'-deoxyadenosine 5'-monophosphate; 3'-AMP, adenosine 3'-monophosphate; dCDP, 2'-deoxycytidine diphosphate; dCMP, 2'-deoxycytidine 5'-monophosphate; AMP, adenosine monophosphate; ACC, 1-aminocyclopropane-1-carboxylic acid; GABA, 4-acetamidobutyric acid; GlcNAc, N-acetyl-D-glucosamine; GlcNAc-1p, N-acetyl- glucosamine-1-phosphate.
